# Safety Investigations of Two Formulations for Vaginal Use Obtained from *Eugenia uniflora* L. Leaves in Female Rats

**DOI:** 10.3390/ph15121567

**Published:** 2022-12-15

**Authors:** Guilherme Donadel, Mariana Dalmagro, João Antonio Berta de Oliveira, Giuliana Zardeto, Mariana Moraes Pinc, Jaqueline Hoscheid, Odair Alberton, Salviano Tramontin Belettini, Ezilda Jacomassi, Arquimedes Gasparotto Junior, Emerson Luiz Botelho Lourenço

**Affiliations:** 1 Laboratory of Preclinical Research of Natural Products, Paranaense University, Umuarama 87502-210, Parana, Brazil; 2 Laboratory of Cardiovascular Pharmacology (LaFaC), Faculty of Health Sciences, Federal University of Grande Dourados, Dourados 79804-970, Mato Grosso do Sul, Brazil

**Keywords:** medicinal plants, pitanga, toxicity

## Abstract

Medicinal plants have great prominence in research into the development of new medicines. *Eugenia uniflora* L. (Myrtaceae) is an edible and medicinal plant with economic value in the northeast region of Brazil. Several preparations from *E. uniflora* leaves and its fruits are employed as a source of nutrients and bioactive compounds. In this study we evaluated the preclinical toxicology of crude extract and vaginal gel obtained from the leaves of *E. uniflora* (5%, 10%, and 15%) aiming to provide safety for its use in the treatment of vulvovaginitis. Both formulations were applied to the vaginal cavity for 14 days. Detailed observations of the vaginal region, including pruritus, swelling, irritation, burning, pain, and vaginal secretion, as well as the estrous cycle were evaluated. On the fifth day, blood samples were obtained from the supraorbital plexus for biochemical and hematological analyses. The animals were subsequently euthanized. All animals underwent necropsy and macroscopic examination of the vaginal mucosa and reproductive system. A histological examination was also performed. No clinically significant changes were detected during the entire experimental period. All biochemical, hematological, or histopathological parameters were within the normal range for the species. The data obtained allow us to suggest that the *E. uniflora* vaginal formulations are safe in this experimental model.

## 1. Introduction

Medicinal plants have been a therapeutic alternative for thousands of years, mainly in Middle Eastern countries and Asia. Historically, several species were used in the treatment of several health disorders, the prevention of epidemics, and in microbial and antifungal control [1,2,3]. Brazil has an excellent tradition in the use of different natural products, due mainly to the large diversity in plant species in their natural biomes [4]. In general, traditional preparations from medicinal plants concentrate many metabolites [5], which, despite their effectiveness, can cause significant adverse effects [6,7,8].

Among the Brazilian species with medicinal properties, *Eugenia uniflora* L. (Myrtaceae) stands out. In Brazil, the fruits of *E. uniflora* are consumed in natura, but their main use is in the industrial and domestic preparation of pulps and juices. The species can also be used in the manufacture of ice cream, refreshments, jellies, liqueurs, and wine [4]. In the 15th century, the species was included in traditional medicine by the Guarani Indians. Popularly, it is used in the form of decoction or infusion in the treatment of hypertension, as well as gastric and digestive disorders. Moreover, the consumption of its leaves and fruits showed anti-inflammatory, antioxidant, analgesic, antibacterial, and antifungal effects [4,5,9,10,11]. Several secondary metabolites were described for *E. uniflora*. Recently, Souza et al. [12] characterized the presence of several flavonoids, tannins, and terpenes in crude extracts and volatile oil from *E. uniflora* leaves, including selina-1,3,7(11)-trien-8-one, oxidoselina-1,3,7(11)-trien-8-one, germacrene B, curzerene, β-caryophyllene, germacrone, and β-elemene, which were related to their pharmacological activities.

Diseases of the genital tract, such as bacterial vaginosis, candidiasis, vulvovaginitis, and chlamydia, affect women of all ages by altering the local microbiota, raising the pH, and causing discharge, edema, and vaginal odor [11,13]. In Brazil, plants such as *Schinus terebinthifolius* Raddi (“Aroeira”), *Stryphnodendron barbatimam* Mart. (“Barbatimão”), and *Anacardium occidentale* L. (“Caju”) showed beneficial properties for the treatment of diseases of the gynecological tracts, being even recommended by the Ministry of Health [14,15]. It is worth noting that many studies with different preparations obtained from medicinal plants showed significant effects against several pathogens including *Staphylococcus aureus*, *Listeria monocytogenes*, *Bacillus subtilis*, *Streptococcus faecalis*, *Staphylococcus epidermidis*, as well as resistant strains of *Candida albicans* [11,16]. Thus, in this study we investigated the pre-clinical toxicological of two vaginal formulations obtained from the leaves of *E. uniflora*, aiming to provide safety for its use in the treatment of vulvovaginitis.

## 2. Results

### 2.1. Clinical and Behavioral Observations

During the 14-day period of experimentation, the animals did not show any clinical or behavioral signs of toxicity, including weight loss (Table 1 and Table 2), piloerection, itching, or secretion or edema in the vaginal region. The estrous cycle was regular in all experimental groups, with 4–5 days (Table 1 and Table 2) of duration and without significant cellular morphological changes.

### 2.2. Relative Organ Weight

Relative weights of organs of the different experimental groups at the end of 14 days of topical vaginal treatment are shown in Table 1 and Table 2. The relative weight of the liver, kidneys, spleen, and reproductive system (ovaries, uterus, and vaginal cavity) did not show any significant change after the application of both formulations when compared to the control groups.

### 2.3. Hematological Parameters

Hematological parameters of the different experimental groups at the end of 14 days of topical vaginal treatment are shown in Table 3 and Table 4. No significant hematological changes were observed among all experimental groups

### 2.4. Biochemical Data

Biochemical parameters of the different experimental groups at the end of 14 days of topical vaginal treatment are shown in Table 5 and Table 6. Statistical analyses did not show any significant lateralization when we compared the groups treated with the different controls used.

### 2.5. Histopathological Analysis

**Figure 1:**
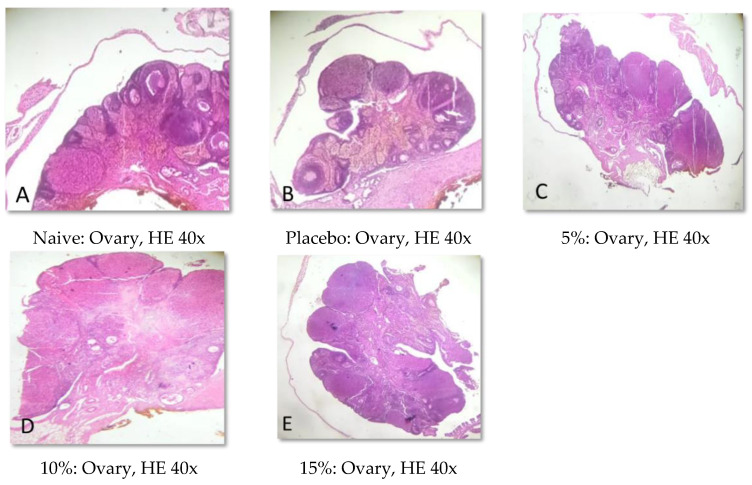
Representative photomicrographs of the ovaries of Wistar rats submitted to vaginal treatment with gel formulation of *Eugenia uniflora* for 14 days. Hematoxylin and Eosin stain.

**Figure 2:**
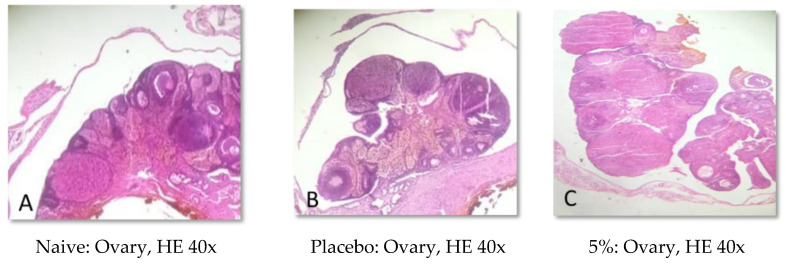

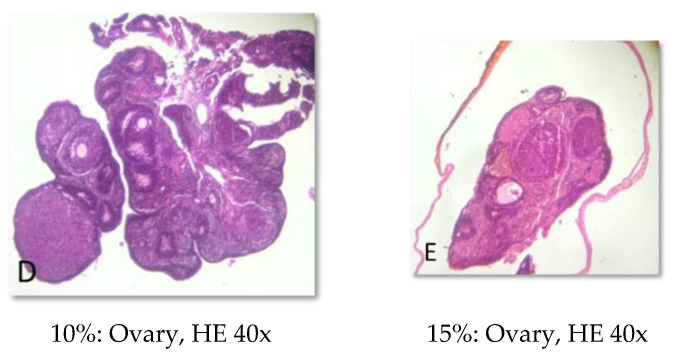
Representative photomicrographs of the ovaries of Wistar rats submitted to vaginal treatment with crude extract of *Eugenia uniflora* for 14 days. Hematoxylin and Eosin stain.

**Figure 3:**
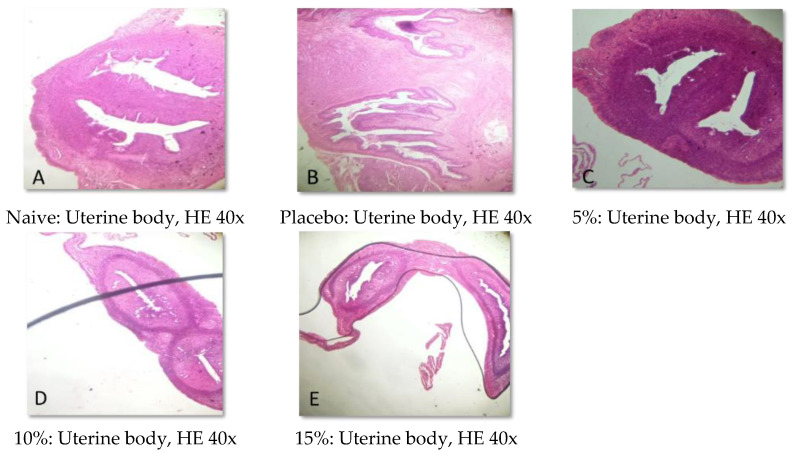
Representative photomicrographs of the uterine body of Wistar rats submitted to vaginal treatment with gel formulation of *Eugenia uniflora* for 14 days. Hematoxylin and Eosin stain.

**Figure 4:**
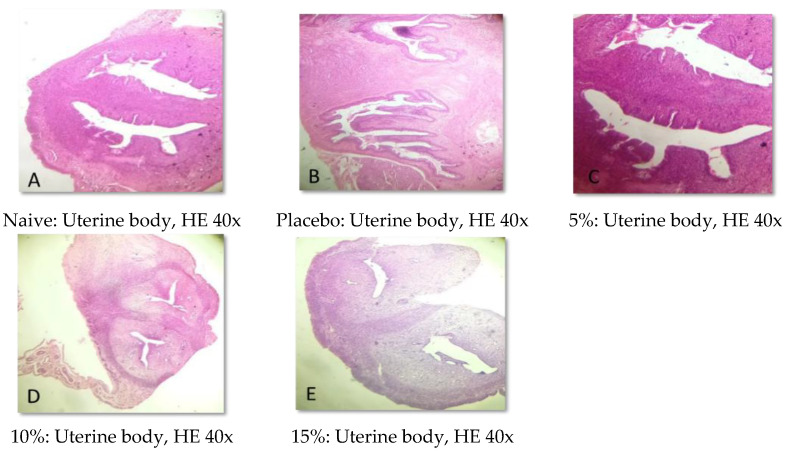
Representative photomicrographs of the uterine body of Wistar rats submitted to vaginal treatment with crude extract of *Eugenia uniflora* for 14 days. Hematoxylin and Eosin stain.

**Figure 5:**
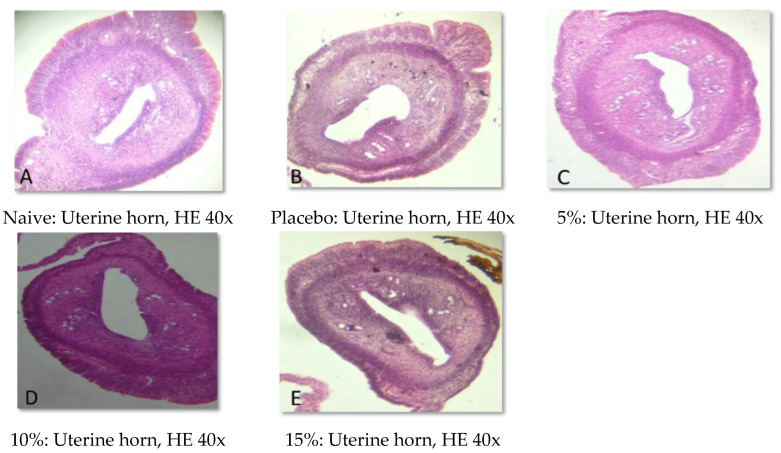
Representative photomicrographs of the uterine horn of Wistar rats submitted to vaginal treatment with gel formulation of *Eugenia uniflora* for 14 days. Hematoxylin and Eosin stain.

**Figure 6:**
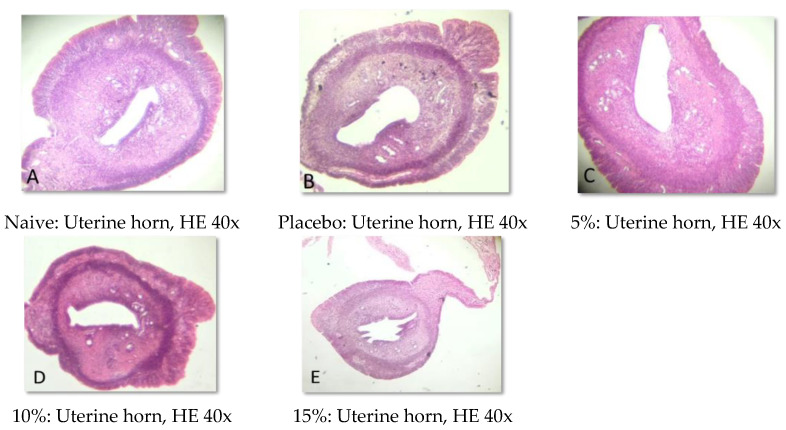
Representative photomicrographs of the uterine horn of Wistar rats submitted to vaginal treatment with crude extract of *Eugenia uniflora* for 14 days. Hematoxylin and Eosin stain.

Representative photomicrographs of ovaries, the uterine body, and the uterine horn are presented in Figure 1, Figure 2, Figure 3, Figure 4, Figure 5 and Figure 6. No significant structural changes were observed between all experimental groups, including apoptosis, necrosis, inflammation, or cellular disarrangement.

## 3. Discussion

Previous studies have shown several beneficial activities of different formulations of *Eugenia uniflora*, including bactericidal, anti-inflammatory, antifungal, and antioxidant effects [4,5,9,10,11,17,18]. Thus, due to the perspective of its topical application in the vaginal region, safety studies are necessary to prove that the extract does not induce deleterious effects in the application site, as well as changes in clinical parameters and in the structures of hormone-responsive organs. In our study, different formulations obtained from *E. uniflora* leaves did not present any systemic clinical signs of toxicity, nor did they induce local edema, pruritus, pain, secretion, bleeding, or mucous plug in the vaginal region after 14 days of treatment.

The female reproductive system has excellent blood irrigation due to high local vascularization, which can significantly facilitate the systemic absorption of numerous locally applied drugs [19,20]. Therefore, safety studies of vaginal formulations comprise, in addition to the direct assessment of the site of administration, a set of parameters that may indicate signs of systemic toxicity. Among the data to be evaluated is the relative weight of the organs, which may increase under conditions of chemical or metabolic stress [21,22]. In addition, serum markers of the hepatic, pancreatic, renal, and endocrine functions may indicate biochemical changes resulting from aggression or work overload for the liver, pancreas, kidneys, as well as the hypothalamic–pituitary–ovarian axis [23,24]. In addition, the evaluation of blood cell production is also worth mentioning, as highly toxic compounds can affect bone marrow and hematopoiesis [21,22]. Both the crude extract and the vaginal gel obtained from *E. uniflora* did not cause any significant systemic changes, as all physical, hematological, and biochemical markers as well as the female sex hormones remained unchanged.

In addition to evaluating the visible effects on the vaginal mucosa, there was a concern about a possible change in vaginal, ovarian, and uterine morphology. In all reproductive organs evaluated, we did not identify any significant morphological changes that could indicate signs of inflammation, degeneration, apoptosis, necrosis, or any adaptation disorders, suggesting a maintained anatomical structure when compared to animals that did not receive any treatment or that were treated only with the vehicle.

In Brazil, several medicinal species are popularly used for the treatment of vulvovaginitis. One of the most relevant is *Stryphnodendron* spp., popularly known as “barbatimão” [25]. In fact, several vaginal pathogenic yeasts can be treated with the stem bark of this species [26]. Despite the benefits, seed extracts of *S. adstringens* and *S. polyphyllum* showed an abortive effect on female rats [27]. In this sense, it is imperative that species popularly used for the treatment of vulvovaginitis undergo a rigorous safety assessment.

Data are available on the toxicological evaluation of different preparations of *E. uniflora*. Assunção Ferreira et al. [22] performed acute and repeated dose toxicity studies and a genotoxicity evaluation with the aqueous extract obtained from *E. uniflora* leaves. This preparation, rich in phenolic compounds such as gallic acid, ellagic acid, and myricetin, showed non-toxic and non-genotoxic effects in rodents. In addition, the hepatoprotective effects of the ethyl acetate extract from *E. uniflora* leaves have already been described, which may indicate additional clinical aspects of the low toxicity of the species [23]. Thus, our study complements the scientific data on the safety of *E. uniflora* preparations and helps to form a knowledge base for the development of new herbal medicine.

## 4. Materials and Methods

### 4.1. Plant Material and Crude Extract Preparation

Leaves of *E. uniflora* were collected and identified by the Instituto Agronômico de Pernambuco (IPA). The crude extract was obtained by the Herbarium Pharmaceutical Laboratory in Phytomedicine (Colombo, Paraná, Brazil). Initially, 10 g of the leaf powder was turbo-extracted with 100 mL of acetone: water (7:3, *v/v*) in four five-minute rounds. The resulting extract was filtered and concentrated by rotary evaporation at 40 °C and 150 rpm (Laborota 4000, Heidolph). The concentrate was subjected to lyophilization at −64 °C and 0.006 mBar (ALPHA 1-2 LDplus, Fisher Scientific, Illkirch-Graffenstaden, France). The hydroacetonic extract was stored at 4 °C until the experiments.

### 4.2. Vaginal Gel Obtention

The vaginal gel was obtained by the Herbarium Pharmaceutical Laboratory in Phytomedicine (Colombo, Paraná, Brazil) at concentrations of 5%, 10%, and 15%. The excipients used in the formulation are in accordance with the Brazilian Pharmacopeia and include polyacrylamide, C13-14 isoalkanes, lauryl alcohol ethoxylate 7 OE, paraben, imidazolidinyl urea at 50%, and purified water. These concentrations were determined according to the pharmaceutical preparations found in the vaginal gels based on *Schinus terebinthifolia* Raddi (“aroeira”), *Stryphnodendron adstringens* (Mart.) (“barbatimão”), and *Melaleuca alternifolia* Cheel (“melaleuca”), which are already available on the Brazilian market.

### 4.3. Pharmacological Assays

#### 4.3.1. Animals

Fifty-six female Wistar rats (90 to 100 days old) were acquired from the vivarium of the Federal University of Grande Dourados (UFGD). The animals were kept in the vivarium at Paranaense University (UNIPAR) in rooms with a constant temperature (22 ± 2 °C) and a light/dark phase of 12 h (lights on from 7 a.m. to 7 p.m.). The animals were divided into eight groups of seven animals and received water and food ad libitum. All procedures were in accordance with the guidelines of the National Council for the Control of Animal Experimentation (CONCEA) and were approved by the Ethics Committee in Research Involving Animal Experimentation of UNIPAR (AUTH number 38108—25 May 2020).

#### 4.3.2. Experimental Design

Initially, the animals were divided into eight experimental groups and received the following treatments:

Group 1: Naive: Animals that were not exposed to treatment.

Group 2: Placebo: Animals that were exposed to the vehicle used in the formulation of the vaginal gel.

Group 3: Vaginal gel 5%: Animals that were exposed to the vaginal gel of *E. uniflora* at 5%.

Group 4: Vaginal gel 10%: Animals that were exposed to the vaginal gel of *E. uniflora* at 10%.

Group 5: Vaginal gel 15%: Animals that were exposed to the vaginal gel of *E. uniflora* at 15%.

Group 6: Crude extract 5%: Animals that were exposed to the crude extract of *E. uniflora* at 5%.

Group 7: Crude extract 10%: Animals that were exposed to the crude extract of *E. uniflora* at 10%.

Group 8: Crude extract 15%: Animals that were exposed to the crude extract of *E. uniflora* at 15%.

The vaginal gel and crude extract of *E. uniflora* were applied over the vaginal cavity, once a day (with the aid of a swab), for 14 days. The crude extract was resuspended in distilled water at the time of application.

#### 4.3.3. Clinical and Behavioral Observations

Daily clinical evaluations of the vaginal region (pruritus, swelling, irritation, burning, pain, and vaginal secretion) were performed. Body weight was determined prior to the beginning of the experiments and on the day of euthanasia. Vaginal smears were performed before the beginning of the treatments and every five days for the entire experimental period. Vaginal lavage was obtained with the aid of a micropipette through vaginal washes with 50 µL of distilled water. The evaluation was performed under optical microscopy (200×). On the morning of the 15th day, the animals were euthanized by deepening anesthetic with Isoflurane at 5% followed by decapitation.

#### 4.3.4. Biochemical Analysis

Approximately 5 mL of blood was collected (by the decapitation method) into tubules with a clotting agent and a polymer gel for serum separation The biochemical parameters analyzed were: urea, creatinine, total protein, albumin, globulins, aspartate amino transferase (AST), alanine amino transferase (ALT), gamma glutamyl transpeptidase (GGT), alkaline phosphatase (AP), total bilirubin (TB), thyroid stimulating hormone (TSH), free T3, lipid profile (HDL-cholesterol, LDL-cholesterol, VLDL-cholesterol, total cholesterol (TC), and triglycerides (TG)), progesterone, and estradiol. Analyses were performed on an automated biochemical analyzer (Selectra E, Vital Scientific, The Netherlands) [28].

#### 4.3.5. Hematological Investigation

Another sample of 3 mL of blood was collected (by the decapitation method) into tubes containing K2EDTA. The samples were properly homogenized and stored under refrigeration (about 4 °C) until processing. The total number of red blood cells (mm^3^), hemoglobin (g/dL), hematocrit (%), mean corpuscular volume (MCV, fL), mean corpuscular hemoglobin (MCM, pG), mean corpuscular hemoglobin concentration (HCCM, g/dL), platelets (mm^3^), monocytes (mm^3^), segmented (mm^3^), lymphocytes (mm^3^), and eosinophils (mm^3^) was measured in an automated hematology analyzer (Abbott Cell Dyn 3500, Abbott Diagnostics, Irving, TX, USA) [28].

#### 4.3.6. Macroscopic Evaluation and Relative Organ Weight

After the euthanasia, the liver, spleen, kidneys, and reproductive system (ovaries, uterus, and vaginal cavity) were removed and submitted to necropsy. After we had determined the absolute weight of each organ, the relative weight was calculated by dividing each animal’s organ weight by their body weight × 100.

#### 4.3.7. Histopathological Analysis

Samples of the uterus and ovaries were collected, fixed in 10% formalin solution, dehydrated, diaphanized, paraffinized, and sectioned to a thickness of 4 µm in a Leica^®^ semi-automatic microtome. The sections were stained with hematoxylin and eosin staining and evaluated by light microscopy.

### 4.4. Statistical Analysis

The results are shown as mean ± standard error of the mean (SEM) of seven rats per group. Parametric data were analyzed by one-way analysis of variance (ANOVA) and differences between groups were evaluated following the Bonferroni correction. Statistical analysis was performed using a GraphPad Prism version 9.4.1 for macOS (GraphPad Prism, San Diego, CA, USA). A *p*-value less than 0.05 was considered statistically significant.

## 5. Conclusions

The data obtained allow us to suggest that the *E. uniflora* vaginal formulations are safe after short-term application in female rats and can support the development of new herbal medicine in the future. Further studies will be needed to investigate the efficacy and safety of the species after prolonged use.

## Figures and Tables

**Table 1:** Body weight, length of estrous cycle, and relative organ weight of the different experimental groups at the end of 14 days of topical vaginal treatment with gel formulation (5, 10 and 15%) from *E. uniflora*.

Parameter	Naive	Placebo	5%	10%	15%
Body weight (g)	218 ± 6.3	221 ± 7.2	219 ± 6.9	220 ± 7.7	222 ± 7.1
Estrous cycle (days)	4.7 ± 0.3	4.6 ± 0.2	4.6 ± 0.3	4.8 ± 0.3	4.7 ± 0.2
Liver (%)	3.79 ± 0.11	3.52 ± 0.10	3.70 ± 0.05	3.51 ± 0.12	3.32 ± 0.20
Spleen (%)	0.25 ± 0.01	0.24 ± 0.01	0.25 ± 0.01	0.24 ± 0.01	0.24 ± 0.01
Kidneys (%)	0.42 ± 0.01	0.41 ± 0.01	0.40 ± 0.01	0.39 ± 0.01	0.41 ± 0.02
Reproductive organs (%)	0.22 ± 0.02	0.22 ± 0.02	0.19 ± 0.01	0.23 ± 0.01	0.24 ± 0.03

Statistical analyses were performed using one–way ANOVA followed by Bonferroni’s post-hoc test. Values are expressed as mean ± SEM (standard error of the mean). *n* = seven rats per group.

**Table 2:** Body weight, length of estrous cycle, and relative organ weight of the different experimental groups at the end of 14 days of topical vaginal treatment with crude extract (5, 10 and 15%) from *E. uniflora*.

Parameter	Naive	Placebo	5%	10%	15%
Body weight (g)	220 ± 5.2	219 ± 6.1	221 ± 7.0	219 ± 6.4	219 ± 6.6
Estrous cycle (days)	4.6 ± 0.3	4.7 ± 0.3	4.6 ± 0.2	4.7 ± 0.3	4.8 ± 0.2
Liver (%)	3.79 ± 0.11	3.52 ± 0.10	3.61 ± 0.14	3.44 ± 0.07	3.87 ± 0.12
Spleen (%)	0.25 ± 0.01	0.24 ± 0.01	0.23 ± 0.01	0.25 ± 0.01	0.24 ± 0.01
Kidneys (%)	0.42 ± 0.01	0.41 ± 0.01	0.40 ± 0.01	0.42 ± 0.02	0.39 ± 0.01
Reproductive organs (%)	0.22 ± 0.02	0.22 ± 0.02	0.21 ± 0.01	0.22 ± 0.02	0.20 ± 0.01

Statistical analyses were performed using one–way ANOVA followed by Bonferroni’s post-hoc test. Values are expressed as mean ± SEM (standard error of the mean). *n* = seven rats per group.

**Table 3:** Hematological parameters of the different experimental groups at the end of 14 days of topical vaginal treatment with gel formulation (5, 10 and 15%) from *E. uniflora*.

Parameter	Naive	Placebo	5%	10%	15%
Red blood cells (mm^3^)	3.7 ± 0.9	5.3 ± 0.2	3.7 ± 0.9	5.1 ± 0.1	4.0 ±1.0
Hemoglobin (g/dL)	7.9 ± 2.0	11.6 ± 0.1	8.3 ± 2.1	11.7 ± 0.3	8.5 ± 2.2
Hematocrit (%)	21.6 ± 5.5	30.1 ± 5.1	22.1 ± 5.8	29.6 ± 4.6	22.4 ± 5.5
MCV (µm^3^)	41.5 ± 10.4	56.4 ± 8.8	41.5 ± 10.7	57.5 ± 9.5	40.1 ± 10.0
HCM (pg)	15.1 ± 3.4	21.9 ± 0.5	15.8 ± 4.0	22.8 ± 4.6	15.4 ± 4.2
CHCM (g/dL)	26.1 ± 6.1	34.8 ± 7.8	27.0 ± 6.9	39.7 ± 8.0	27.5 ± 7.4
Platelets (mm^3^)	241.5 ± 65.2	277.0 ± 56.4	255.6 ± 69.5	294.3 ± 49.5	239.3 ± 64.0
Monocytes (mm^3^)	142.1 ± 40.0	105.1 ± 42.0	120.3 ± 76.4	154.4 ±39.9	128.1 ± 61.0
Segmented (mm^3^)	1448.2 ± 465.0	1214.2 ± 446.0	1102.90 ± 278.5	1397.1 ± 352.8	1121.2 ± 297.3
Lymphocytes (mm^3^)	1904.1 ± 605.0	2390.3 ± 661.3	2278.2 ± 854.1	1606.2 ± 631.0	1697.3± 752.2
Eosinophils (mm^3^)	3.2 ± 2.2	3.7 ± 2.6	3.14 ± 2.1	0	0

Statistical analyses were performed using one–way ANOVA followed by Bonferroni’s post-hoc test. Values are expressed as mean ± SEM (standard error of the mean). *n* = seven rats per group.

**Table 4:** Hematological parameters of the different experimental groups at the end of 14 days of topical vaginal treatment with crude extract (5, 10 and 15%) from *E. uniflora*.

Parameter	Naive	Placebo	5%	10%	15%
Red blood cells (mm^3^)	3.7 ± 0.9	5.3 ± 0.5	5.2 ± 0.1	4.5 ± 0.7	5.0 ± 0.8
Hemoglobin (g/dL)	11.2 ± 2.0	11.6 ± 1.3	12.1 ± 2.3	10.3 ± 1.7	11.0 ± 1.8
Hematocrit (%)	27.9 ± 5.5	30.1 ± 1.3	30.1 ± 2.7	25.6 ± 2.3	26.6 ± 4.4
MCV (µm^3^)	41.7 ± 7.4	46.4 ± 8.8	57.0 ± 9.9	48.1 ± 8.1	45.4 ± 7.5
HCM (pg)	17.9 ± 3.4	21.9 ± 3.5	23.0 ± 2.6	19.4 ± 3.3	18.7 ± 3.1
CHCM (g/dL)	36.6 ± 6.8	38.8 ± 6.8	40.4 ± 5.9	34.7 ± 6.0	35.5 ± 5.9
Platelets (mm^3^)	281.0 ± 65.8	317.0 ± 46.4	293.6 ± 45.3	291.4 ± 56.8	300.0 ± 57.9
Monocytes (mm^3^)	142.1 ± 20.3	151.2 ± 28.0	145.1 ± 32.8	139.2 ± 29.1	135.2 ± 36.1
Segmented (mm^3^)	1448.1 ± 365.5	1214.1 ± 346.0	1268.2 ± 310.7	1290.4 ± 342.7	1263.6 ± 200.8
Lymphocytes (mm^3^)	3904.2 ± 605.1	4190.1 ± 661.3	3913.1 ± 599.7	3942.1 ± 570.3	4128.1 ± 622.9
Eosinophils (mm^3^)	2.2 ± 1.2	3.1 ± 2.6	0	2.4 ± 1.4	0

Statistical analyses were performed using one–way ANOVA followed by Bonferroni’s post-hoc test. Values are expressed as mean ± SEM (standard error of the mean). *n* = seven rats per group.

**Table 5:** Biochemical parameters of the different experimental groups at the end of 14 days of topical vaginal treatment with gel formulation (5, 10 and 15%) from *E. uniflora*.

Parameter	Naive	Placebo	5%	10%	15%
Total protein (g/dL)	6.14 ± 0.51	6.33 ± 1.11	6.32 ± 0.55	6.34 ± 0.62	6.31 ± 0.55
Albumin (g/dL)	4.11 ± 0.32	3.99 ± 0.66	3.87 ± 0.78	4.01 ± 0.40	4.04 ± 0.69
Globulin (g/dL)	2.33 ± 0.23	2.23 ± 0.33	2.40 ± 0.29	2.42 ± 0.39	2.28 ± 0.39
AST (U/L)	126.73 ± 41.23	121.66 ± 55.42	130.22 ± 41.23	120.12 ± 41.74	131.33 ± 41.23
ALT (U/L)	55.57 ± 11.20	61.12 ± 8.30	51.5 ± 9.98	49.15 ± 10.53	59.17 ± 11.30
GGT (U/L)	1.52 ± 0.54	1.47 ± 0.58	1.55 ± 0.69	1.67 ± 0.66	1.62 ± 0.64
AP (U/L)	104.15 ± 55.22	112.34 ± 47.49	114.6 ± 42.27	111.13 ± 54.45	115.15 ± 75.12
TB (mg/dL)	0.047 ± 0.017	0.046 ± 0.017	0.049 ± 0.016	0.050 ± 0.020	0.049 ± 0.019
TG (mg/dL)	77.12 ± 7.01	71.22 ± 8.12	69.22 ± 9.03	73.11 ± 8.76	69.72 ± 9.11
TC (mg/dL)	60.17 ± 11.02	61.57 ± 10.04	59.11 ± 9.02	60.25 ± 8.12	68.17 ± 10.02
HDL-C (mg/dL)	28.22 ± 6.14	30.29 ± 7.11	32.37 ± 9.11	30.71 ± 9.21	29.11 ± 8.84
VLDL-C (mg/dL)	13.12 ± 3.12	12.11 ± 3.01	11.44 ± 4.01	11.04 ± 3.42	10.82 ± 3.99
LDL-C (mg/dL)	21.11 ± 3.23	25.33 ± 4.11	26.13 ± 5.03	24.21 ± 3.99	22.22 ± 2.83
Creatinine (mg/dL)	0.33 ± 0.08	0.35 ± 0.09	0.39 ± 0.06	0.39 ± 0.07	0.34 ± 0.08
Urea (mg/dL)	50.01 ± 8.31	48.12 ± 6.99	50.99 ± 7.78	53.07 ± 9.11	52.06 ± 10.40
Progesterone (pg/mL)	28.06 ± 7.34	30.11 ± 6.16	26.99 ± 8.79	33.31 ± 9.32	29.11 ± 7.65
Estradiol (pg/mL)	47.22 ± 11.23	54.55 ± 12.21	55.33 ± 10.09	50.12 ± 10.21	45.22 ± 11.33
Free T3 (ng/mL)	0.52 ± 0.11	0.55 ± 0.10	0.53 ± 0.09	0.48 ± 0.09	0.51 ± 0.11
TSH (ng/mL)	4.71 ± 0.70	5.02 ± 0.72	4.99 ± 0.55	5.11 ± 0.99	4.95 ± 0.79

Statistical analyses were performed using one–way ANOVA followed by Bonferroni’s post-hoc test. Values are expressed as mean ± SEM (standard error of the mean). *n* = seven rats per group.

**Table 6:** Biochemical parameters of the different experimental groups at the end of 14 days of topical vaginal treatment with crude extract (5, 10 and 15%) from *E. uniflora*.

Parameter	Naive	Placebo	5%	10%	15%
Total protein (g/dL)	6.14 ± 0.51	6.33 ± 1.11	6.33 ± 0.99	6.51 ± 0.97	6.61 ± 0.66
Albumin (g/dL)	4.11 ± 0.32	3.99 ± 0.66	3.92 ± 0.55	3.87 ± 0.77	4.02 ± 0.44
Globulin (g/dL)	2.33 ± 0.23	2.23 ± 0.33	2.45 ± 0.40	2.37 ± 0.33	2.24 ± 0.41
AST (U/L)	126.73 ± 41.23	121.66 ± 55.42	126.16 ± 56.56	120.42 ± 56.65	141.23 ± 42.31
ALT (U/L)	55.57 ± 11.20	61.12 ± 8.30	53.5 ± 9.98	52.65 ± 10.56	55.77 ± 11.20
GGT (U/L)	1.52 ± 0.54	1.47 ± 0.58	1.55 ± 0.59	1.57 ± 0.61	1.61 ± 0.54
AP (U/L)	104.15 ± 55.22	112.34 ± 47.49	114.10 ± 44.17	110.42 ± 51.15	109.15 ± 49.29
TB (mg/dL)	0.047 ± 0.017	0.046 ± 0.017	0.048 ± 0.019	0.049 ± 0.020	0.047 ± 0.017
TG (mg/dL)	77.12 ± 7.01	71.22 ± 8.12	70.77 ± 8.21	68.99 ± 9.99	68.19 ± 8.89
TC (mg/dL)	60.17 ± 11.02	61.57 ± 10.04	67 ± 8.02	66.15 ± 8.19	69.17 ± 10.22
HDL-C (mg/dL)	28.22 ± 6.14	30.29 ± 7.11	35.66 ± 9.99	32.77 ± 7.54	27.55 ± 5.55
VLDL-C (mg/dL)	13.12 ± 3.12	12.11 ± 3.01	12.14 ± 2.99	13.09 ± 3.99	14.82 ± 4.22
LDL-C (mg/dL)	21.11 ± 3.23	25.33 ± 4.11	24.13 ± 5.02	23.11 ± 4.99	20.92 ± 4.12
Creatinine (mg/dL)	0.33 ± 0.08	0.35 ± 0.09	0.39 ± 0.07	0.37 ± 0.08	0.36 ± 0.09
Urea (mg/dL)	50.01 ± 8.31	48.12 ± 6.99	49.22 ± 8.43	50.07 ± 9.31	52.66 ± 9.66
Progesterone (pg/mL)	28.06 ± 7.34	30.11 ± 6.16	30.22 ± 8.11	33.07 ± 9.31	28.21 ± 8.31
Estradiol (pg/mL)	47.22 ± 11.23	54.55 ± 12.21	52.01 ± 10.99	48.98 ± 8.99	45.22 ± 11.21
Free T3 (ng/mL)	0.52 ± 0.11	0.55 ± 0.10	0.52 ± 0.10	0.52 ± 0.10	0.53 ± 0.11
TSH (ng/mL)	4.71 ± 0.70	5.02 ± 0.72	4.93 ± 0.77	5.01 ± 0.89	4.98 ± 0.88

Statistical analyses were performed using one–way ANOVA followed by Bonferroni’s post-hoc test. Values are expressed as mean ± SEM (standard error of the mean). *n* = seven rats per group.

## Data Availability

Data is contained within the article.
